# The Age Effects on the Cognitive Processes of Intention-Based and Stimulus-Based Actions: An ERP Study

**DOI:** 10.3389/fpsyg.2017.00803

**Published:** 2017-05-29

**Authors:** Ya-Nan Niu, Xinyi Zhu, Juan Li

**Affiliations:** ^1^Center on Aging Psychology, CAS Key Laboratory of Mental Health, Institute of Psychology, Chinese Academy of SciencesBeijing, China; ^2^Department of Psychology, University of Chinese Academy of SciencesBeijing, China; ^3^State Key Laboratory of Brain and Cognitive Science, Institute of Biophysics, Chinese Academy of SciencesBeijing, China

**Keywords:** age effect, motor cognition, intention-based action, stimulus-based action, bidirectional action-effect association, ERPs, associative memory, executive function

## Abstract

The functional decline in action among older adults is caused not only by physical weakness but also by cognitive decline. In this study, we aimed to compare the cognitive effects of age between intention-based and stimulus-based action modes electrophysiologically. Because age-related declines in cognitive function might proceed distinctly according to specific action modes and processes, four specific cognitive processes, *action-effect binding, stimulus-response linkage, action-effect feedback control*, and *effect-action retrieval*, were investigated. We recorded event-related potentials (ERPs) during a modified acquisition-test paradigm in young (mean age = 21, *SD* = 2) and old (mean age = 69, *SD* = 5) groups. A temporal bisection task and a movement pre-cuing task were used during the acquisition and test phases, respectively. Using ERP indices including readiness potential (RP), P3, N2 and contingent negative variation (CNV) to identify these four specific processes for the two action modes, we revealed the effects of age on each ERP index. The results showed similar patterns of waveforms but consistently decreasing amplitudes of all four ERP indices in the old age group compared with the young age group, which indicates not only generally declining functions of action preparation in older adults but also age effects specific to the action modes and processes that might otherwise be mixed together under confounding experimental conditions. Particularly, an interference effect indexed by the differences in the amplitudes of CNV between congruent and incongruent tasks was observed in the young age group, which is consistent with previous behavioral reports. However, this effect was absent in the old age group, indicating a specific age-related deficit in the *effect-action retrieval* process of intention-based action, which might be caused by an age-related deficit in associative memory. In sum, this study investigated the cognitive processes of two action modes from a developmental perspective and suggests the importance of adding associative memory training to interventions for older adults with the aim of improving intention-based action.

## Introduction

The functional decline in action among older adults is caused not only by physical weakness but also by cognitive decline. To date, the effects of healthy aging on cognitive processes of action are not well understood (Onofrj et al., 2001 for a review; Sterr and Dean, 2008; Reuter et al., 2015).

Age-related declines in the prefrontal cortex (PFC) and executive function have been implicated in changes in both the motor preparation and execution phases (Sterr and Dean, 2008; Berchicci et al., 2012; Woods et al., 2015). The possible underlying mechanism involves the compensatory mechanism, overactivation of specific brain areas, and dedifferentiation (see Reuter-Lorenz, 2002 for a review). However, the effect of age on more specific cognitive processes remains unclear. According to the frontal lobe hypothesis (West, 1996, 2000) and reverse theory (Braak et al., 1999), age-related declines in brain areas might proceed distinctly. Thus, the related cognitive processes would be impaired distinctly. Therefore, it is necessary to compare the age effect between different action modes under specific cognitive processes to further understand the effects of age on motor cognition.

Recently, a series of studies investigating motor cognition by comparing intention-based and stimulus-based actions has verified several specific cognitive processes of actions (Elsner and Hommel, 2001; Haggard et al., 2002; Waszak et al., 2005; Keller et al., 2006; Herwig et al., 2007; Herwig, 2015 for a review). Using an acquisition-test paradigm, a bidirectional action-effect association was identified for intention-based action, which included the cognitive processes of *action-effect binding* and *effect-action retrieval*, whereas the cognitive process of *stimulus-response linkage* was observed for stimulus-based action. Interestingly, the *effect-action retrieval* process of intention-based action resembled the retrieval of associative memory (Elsner et al., 2002; Melcher et al., 2008, 2013; Pfister et al., 2014), suggesting that associative memory was also involved in the cognitive processes of action. For older adults, it is well known that associative memory is more vulnerable than other types of memory (e.g., Naveh-Benjamin, 2000; see Yonelinas, 2002 for a review; Rhodes et al., 2008). Therefore, in this study, using certain paradigms, we aimed to investigate the age effects on several specific cognitive processes of actions between young and old age groups, with special interest in the associative retrieval of intention-based action.

In previous studies, the acquisition-test paradigm has been primarily used to reveal differences in the cognitive mode between intention-based and stimulus-based actions (e.g., Elsner and Hommel, 2001; Herwig et al., 2007). Participants were divided into two groups and participated in both the acquisition and test phases. During the acquisition phase, the intention-based group of participants was instructed to make a self-selected keypress (left or right keypress, i.e., action) which was always followed by a certain tone (high or low pitch, i.e., effect), whereas the stimulus-based group were instructed to respond to the stimuli according to prespecified rules, although the response was also followed by certain high- or low-pitch tones. Thus, *action-effect binding* was acquired by intention-based acquisition and *stimulus-linkage* was acquired by stimulus-based acquisition. In the subsequent test phase, participants were asked to perform a rapid keypress for the same tones with a high or low pitch according to either the congruent or incongruent response rule with the acquired linkages. The results showed that the interference effect of reaction time (RT), that is, faster performance on the congruent task compared with incongruent task, was only observed in intention-based action. These results demonstrated a bidirectional action-effect association in intention-based action, that is, the learned *action-effect binding* during acquisition was then retrieved in reverse (i.e., *effect-action retrieval*) during the test phase. The RT performance of *effect-action retrieval* was thus prolonged by the interference from the incongruent task. However, this interference effect was absent in stimulus-based action, suggesting that a mono-directional *stimulus-response linkage* was formed.

As Elsner et al. (2002) suggested, this *effect-action retrieval* observed in intention-based actions resembles the process of associative retrieval of memory, and this conclusion has been supported by PET and fMRI studies revealing activation in both the supplementary motor area (SMA) and medial temporal memory system during either action image or execution tasks (Elsner et al., 2002; Melcher et al., 2008, 2013; Pfister et al., 2014). This *effect-action retrieval*, as well as the age effect on this process, has been seldom investigated using event-related potentials (ERPs); however, several studies have reported the ERP indices of the acquisition phase for intention-based and stimulus-based actions. In these ERP studies (Waszak et al., 2005; Keller et al., 2006), a temporal bisection task was used during the acquisition phase to ensure that the two action modes were measured in a comparable manner when the ERP technique was applied (see Materials and Methods). The results demonstrated that the readiness potential (RP) component, which proceeded slowly and negatively before action execution, reflecting general preparation for voluntary movement (e.g., Shibasaki and Hallett, 2006), was more negative for intention-based acquisition, whereas the P3 component—a positive potential observed approximately 300 ms after stimulus presentation, reflecting the formation of a link between the stimulus evaluation and response selection (e.g., Petruo et al., 2016)—was more positive under stimulus-based conditions. In this study, we will use the time bisection task during the acquisition phase to compare the age effect on specific cognitive processes of intention-based and stimulus-based actions, with *action-effect binding* indexed by RP amplitude and *stimulus-response linkage* indexed by P3 amplitude. In this study, the age effects were predicted to be a decrease in the amplitude of both the RP and P3 components in the old age group because both stimulus processing and motor preparation have been reported to be responsible for the age-related decline of actions (Kolev et al., 2006; Sterr and Dean, 2008; Berchicci et al., 2012; Woods et al., 2015).

In addition to these two cognitive processes during the acquisition phase, another cognitive process, namely *feedback control* of intention-based action, was also included in this study. When a participant performed an intention-based action to fulfill a certain goal, the action effect was predicted and compared with the actual action effect as observed from the external environment using a comparator system (Blakemore et al., 2000 for a review; Hughes and Waszak, 2011; Hughes et al., 2013 for a review). This *feedback control* mechanism has been reported to be associated with the anterior cingulate cortex (ACC) and function as a performance monitoring and adjustment system (Debener et al., 2005). In a recent ERP study (Hughes and Waszak, 2011), this *feedback control* process was observed by the N2 amplitude, which was fronto-centrally distributed within a 150–350 ms time window after the appearance of the action effect, followed immediately by the P3 component. Similarly, in this study, we expected to detect this *feedback control* process based on the N2 amplitude during the acquisition phase in intention-based action. The age effect was predicted to be a decrease in the N2 amplitude in the old age group, especially regarding intention-based action.

In terms of the *effect-action retrieval* process during the test phase, the contingent negative variation (CNV) amplitude in a movement pre-cuing task was used to reflect the action preparation before the action execution. In the movement pre-cuing task, the upcoming movement was informed in advance by the pre-cue, and the amplitude of the CNV tended to increase with the amount of advance information provided by the pre-cue, either because this information enabled more sufficient preparation for the upcoming action (Leuthold et al., 2004) or because additional resources were available for the subjects to complete the task (Falkenstein et al., 2003). In this study, we assumed that, in intention-based action, the CNV amplitude would increase in congruent tasks because the congruent response rule facilitates action preparation, in contrast to incongruent tasks, in which the incongruent response rule interferes with action preparation. Therefore, the difference in the CNV amplitude between congruent and incongruent tasks demonstrates the interference effect of RT in intention-based actions in the young age group, as introduced in previous behavioral studies. Such an interference effect of CNV amplitude would not be observed in stimulus-based action. In our previous study (Niu et al., 2016), this interference effect of the CNV amplitude was absent in intention-based action in healthy older adults even after receiving cognitive training. In this study, we predicted that we would observe this interference effect of the CNV amplitude in the young age group but not in the old age group, which would indicate an age-related impairment on the *effect-retrieval process* in intention-based action. A correlation analysis was conducted between the cognitive performance and CNV amplitude in each task to investigate whether associative memory scores were responsible for this interference effect.

In summary, multiple cognitive processes may be involved in intention-based and stimulus-based actions. As suggested by the frontal lobe hypothesis (West, 1996, 2000) and the reverse theory (Braak et al., 1999), age-related declines occur in distinct brain areas, according to the different cognitive functions and processes. Therefore, it is necessary to investigate the age effect under specific cognitive processes, which might be otherwise mixed in confounding experimental conditions. Using the acquisition-test paradigm and time-bisection task, we aimed to investigate the age effects on actions by comparing specific cognitive processes between intention-based and stimulus-based action modes in young and old age groups. Four specific cognitive processes, *action-effect binding, stimulus-response linkage, action-effect feedback control*, and *effect-action retrieval*, were investigated indexed using the RP, P3, N2, and CNV amplitudes, respectively. Compared with stimulus-based actions, intention-based actions have been suggested to be more evolved and play an important role in daily life (Neumann, 1990; see Herwig, 2015 for a review), which was the main aim of this study. In particular, we wanted to investigate the age effect on the *effect-action retrieval* process in intention-based actions and the potential relationship between action retrieval and associative memory retrieval. We hypothesized that (1) generally declining functions of action preparation in older adults would be revealed by consistently decreasing ERP amplitudes in four cognitive processes of actions; (2) age-related decline would be observed under each specific cognitive process, which might be otherwise mixed and misinterpreted in confounding conditions; (3) particularly, an age effect on *effect-action retrieval* process of intention-based action would be revealed via the interference effect of the CNV amplitude between congruent and incongruent tasks, which would be observed in the young age group and be absent in the old age group, possibly due to the age-related deficit of associative memory.

## Materials and Methods

### Participants

Thirty-six healthy subjects participated in the study, including 18 young subjects (10 women and 8 men) aged 19–24 years (mean age = 21, *SD* = 2) and 18 elderly subjects (9 women and 9 men) aged 61–74 years (mean age = 69, *SD* = 5). The age groups were matched for years of education (young subjects: 14 years, *SD* = 1; elderly: 13 years, *SD* = 3).

All of the subjects were recruited through advertisements placed in universities and communities near the Institute of Psychology, Chinese Academy of Sciences. The young subjects were all undergraduates that were active in the university. All of the subjects were right-handed, had no history of severe psychiatric or neurological disease, did not use drugs that might adversely affect cognition (i.e., benzodiazepines or antipsychotics), had normal or corrected-to-normal vision and audition, and scored ≤16 on the Center for Epidemiologic Studies Depression Scale (CES-D, Roberts and Vernon, 1983). The Mini Mental State Examination (MMSE, Folstein et al., 1975) was used as a screening test for dementia, and all elderly subjects included in this study scored ≥27 out of 30 possible points. Both the young and elderly subjects were also administered neuropsychological tests, including the Paired Associative Learning Test (PALT, Xu and Wu, 1986), a test of associative memory of word pairs, and Trail Making Test A and B (TMT, Reitan, 1955), to test executive function. The test results are shown in **Table 1**.

**Table 1:** Neuropsychological test scores for the two age groups.

	Young (*n* = 18) *Mean* (*SD*)	Old (*n* = 18) *Mean* (*SD*)	*P*-values (*t*-test)
MMSE	–	27.78 (1.26)	–
CESD	9.39 (3.47)	12.17 (2.96)	0.014
PALT	19.89 (4.90)	11.78 (8.23)	0.002
TMT A^a^	20.86 (6.17)	40.10 (17.14)	0.001
TMT B^a^	27.55 (6.87)	62.51 (27.85)	<0.001

*PALT, paired associative learning test; TMT, trail making test. ^a^Lower scores indicate better performance.*

All of the subjects were naïve to the purpose of the experiment and were financially reimbursed for their participation. The study was approved by the Ethics Committee of the Institute of Psychology, Chinese Academy of Sciences. Written informed consent was procured from all of the subjects prior to participation.

All subjects participated in both the acquisition and test phases. For the analysis of the RP, N2 and P3 components during the acquisition phase, 18 young subjects and 18 elderly subjects were used. For the analysis of CNV components during the test phase, the subjects in each age group were divided into two subgroups that performed congruent or incongruent tasks. For the young age group, 9 subjects (5 women, mean age = 22, *SD* = 1) performed congruent tasks, and 9 subjects (5 women, mean age = 21, *SD* = 1) performed incongruent tasks. For the old age group, 8 subjects (2 women, mean age = 70, *SD* = 4) performed congruent tasks, and 10 subjects (7 women, mean age = 69, *SD* = 5) performed incongruent tasks. The order of the intention-/stimulus-based acquisition and the congruent/incongruent tasks was counterbalanced for each group.

### Apparatus and Stimuli

The subjects were seated in a relaxed position on a comfortable chair in front of a computer screen placed on a table 75 cm away from the subjects’ eyes in a dimly lit, sound-attenuated and electrically isolated room. The two response keys were located on a keyboard and were separated by a horizontal distance of 45 mm. The fixation site was a white cross (“+”) depicted on a black background at a visual angle of 0.8° × 0.8°. The possible visual stimuli were the letters A, T, O, and X presented in white (height: 1.7°) against a black background at the center of the computer screen. During the test phase, a red asterisk (“^∗^”) with a visual angle of 0.8° × 0.8° presented centrally in the screen was used as the imperative signal for a keypress response. At the beginning of each block, an auditory pacing signal composed of sine tones with a frequency of 600 Hz and a duration of 100 ms was presented at a comfortable volume. The stimulus presentation and data collection were performed using E-prime (Version 2.0) software in a Windows XP system.

### Tasks and Procedures

**FIGURE 1:**
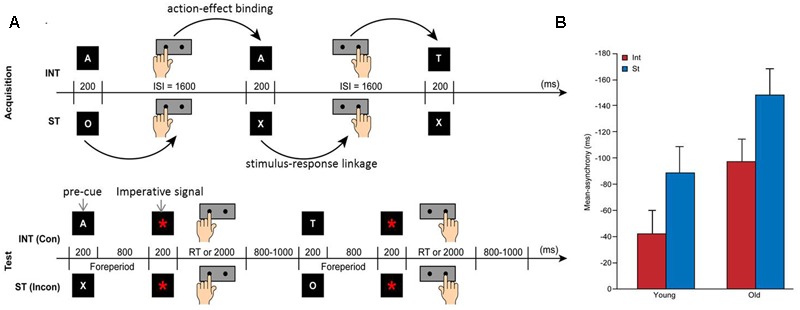
**Experiment design (A)** and behavioral results **(B)**. **(A)** Experimental procedure for the acquisition (upper) and test (lower) phases showing the trial sequence of intention-based (INT) and stimulus-based (ST) actions in congruent (Con) and incongruent (Incon) tasks. ISI, Inter-Stimulus Interval (adapted from Niu et al., 2016). **(B)** Mean asynchrony of temporal bisection for intention-based (red bars) and stimulus-based (blue bars) actions in both age groups during the acquisition phase. The error bars represent the standard errors.

The experiment lasted approximately 3 h, including the time required for instruction and practicing, fixing the electroencephalogram (EEG) electrodes to the scalp, and completing the tasks. Each subject completed four experimental sessions, including the acquisition and test phases, for both intention-based and stimulus-based actions. **Figure 1A** showed the tasks and procedures used in this study. During the acquisition phase, all of the subjects performed intention-based and stimulus-based acquisition with a temporal bisection task to acquire the action-effect and stimulus-response pairs, respectively. During the subsequent test phase, the subjects in each age group were divided into two subgroups to perform a movement pre-cuing task in which the keypress was determined by the letters acquired during acquisition phase. Half of the subjects performed a congruent task in which the keypress was determined by the congruent letter they acquired, whereas the remaining half performed an incongruent task in which the keypress was determined by a letter incongruent with the letter they acquired.

#### Acquisition Phase

The temporal bisection task used during the acquisition phase was adapted from Keller et al. (2006). Before the formal experiment, subjects performed practice trials for time bisection *per se*, which was a pure timing task. This task required them to press either of the two response keys to bisect a 1,600 ms inter-stimulus interval (ISI) with a fixation cross (“+”) presented at the center of the screen. Each presentation of a 1,600-ms ISI was indicated by a centrally placed number eight (“8”) that appeared on the screen for 200 ms. There were 20 trials in each practice block, after which the subjects were informed of their timing performance to indicate whether their responses were relatively fast or slow within 350 ms of the exact bisection time point. Subjects entered the formal experiment when they reported that they were ready for the task and when their performance was adequate, that is, the 80% criterion of response accuracy rates was reached.

The subjects were then fixed with EEG electrodes on their scalps before the screen and performed both intention-based and stimulus-based acquisition sessions recorded by EEG. **Figure 1A** (upper panel) showed the task procedure during acquisition phase. For the intention-based acquisition, the subjects were asked to press the left or right key in a self-selected way to bisect the 1600-ms ISI. Each key press produced an “action effect” in which a letter (i.e., A, T, O, or X) appeared for 200 ms at the end of each ISI. For example, the self-selection of the left keypress determined the appearance of the letter A, and the self-selection of the right keypress determined the appearance of the letter T. The subjects were instructed to attempt to produce a random sequence of letters, as if they were tossing a coin, rather than produce a sequence of a certain order. For the stimulus-based acquisition, the subjects were asked to perform the bisection time task by pressing the left or right key according to the appearance of a letter. For example, the appearance of the letter O signaled a left keypress, and the appearance of the letter X signaled the right keypress. These letters were presented in a randomized and counterbalanced sequence. To eliminate carry-over effects, different letters were presented for the same subject across the two actions.

Subjects could take a break between blocks, which contained 40 trials. An auditory pacing signal was presented in the first 10 trials of each block to indicate the true ISI midpoint and assist subjects with their timing performance. For the remaining 30 trials, the subjects performed the time bisection without the signal. To calculate the correct responses, the time duration of ±350 ms of the true bisection point of 800 ms was examined. Responses that fell within this time duration were considered correct. The blocks were repeated until 200 correct responses (excluding auditory signaled trials) were achieved for each action mode. Thus, sufficient trials were ensured for the ERP average under each condition.

#### Test Phase

The subjects entered the test phase when the acquisition tasks of both the intention-based and stimulus-based actions were complete. **Figure 1A** (lower panel) showed the task procedure during test phase. During the test phase, one subgroup performed a congruent task, and the other subgroup performed an incongruent task. Two sessions of the movement pre-cuing task were administered in which the previously acquired two pairs of letters (e.g., A and T, O and X) were used as pre-cues to signal the subsequent keypress. After the presentation of the fixation cross (“+”) for 200 ms, the pre-cue (i.e., A, T, O, or X) appeared in the center of the screen for the next 200 ms to indicate a left or right keypress. For the congruent task, subjects were asked to press the left or right key according to the congruent rule learned during the acquisition phase. For example, subjects who had acquired left keypress→A/right keypress→T were then required to respond to A with a left keypress and to T with a right keypress. For the incongruent task, subjects were asked to perform the opposite keypresses. For example, subjects who had acquired O→left keypress/X→right keypress were then required to respond to O with a right keypress and to X with a left keypress.

To ensure sufficient motor preparation before the motor response (Falkenstein et al., 2003; Leuthold et al., 2004), subjects were instructed not to respond immediately to the pre-cue but to withhold their response until the imperative signal (a red asterisk “^∗^”) appeared on the center of the screen 1000 ms after pre-cue presentation. After the imperative signal appeared for 200 ms, the response period lasted for 2000 ms. Responses that fell beyond this 2200-ms time period were classified as incorrect. The next trial began after the presentation of a black screen for a random duration of 800–1000 ms. Each test session consisted of 200 trials. The experiment ended after the two test sessions were completed.

### Behavioral Data Analysis

The first 20 trials of each block were discarded from the analysis (i.e., the 10 trials in which the pacing signal was presented and the following 10 trials). Data analysis was performed on the correct responses for the remaining trials. Response accuracy during the acquisition task was calculated by measuring the asynchrony (in ms) of each keypress from the true bisection point of 800 ms. In other words, the asynchrony of each keypress was measured by subtracting the actual response time from 800 ms. Asynchronies that fell within ± 350 ms of the true bisection point were considered correct responses and were then averaged according to action modes and age groups. Repeated-measures analysis of variance (ANOVA) considering the action modes (intention-based or stimulus-based action) as a within-subject factor and age group (young or old) as a between-subject factor was performed. Significant interactions were analyzed using simple effects models. The significance level was set at *p* = 0.05 for all of the analyses.

### Electroencephalogram Recording and Data Analysis

The Neuroscan EEG system (Neuroscan Inc.) continuously recorded EEG signals from 64 scalp electrodes based on the international 10–20 system, with two electrodes placed on the left and right mastoids. The left mastoid served as an online reference. Eye blinks and movements were recorded with two pairs of electrodes as vertical and horizontal electrooculograms (VEOG and HEOG, respectively): one pair placed approximately 1 cm above and below the left eye, and the other pair placed approximately 1 cm lateral to the outer canthi of both eyes. The EEG data were amplified with a band-pass filter of 0.05–100 Hz and were digitized at 500 Hz. Inter-electrode impedances were maintained below 5 kΩ.

Off-line analysis of EEG data was performed, and all data were re-referenced to the average of both mastoids. Ocular artifacts were removed using a regression procedure (Semlitsch et al., 1986). A band-pass filter with a low-pass filter with a cutoff frequency of 30 Hz was used. Epochs of RP were time-locked to the onset of a keypress response and segmented from -900 ms before keypress response to 400 ms after keypress response, with -900 to -800 ms (i.e., the very beginning 100 ms of RP epoch) serving as the baseline period. Epochs of 700 ms (including the pre-stimulus baseline time of 100 ms) were extracted for N2/P3, and 2,200 ms segments (including the pre-stimulus baseline time of 200 ms) were extracted for CNV; both were time-locked to the onset of the stimulus (i.e., A, T, O, or X). Epochs exceeding ± 100 μV were considered artifacts and were rejected from further analysis for both age groups.

During the acquisition phase, RP was measured as the mean amplitude at electrodes F3, Fz, F4, C3, Cz, C4, P3, Pz, and P4 within a time window of -400–0 ms before keypress response because these signals reflect general motor preparation prior to action (Shibasaki and Hallett, 2006). The mean amplitude of N2 was measured within the time window of 300–400 ms over 9 fronto-central sites (F3, Fz, F4, FC3, FCz, FC4, C3, Cz, and C4). The mean amplitude of P3 was measured within 400–500 ms over centro-parietal sites (C3, Cz, C4, CP3, CPz, CP4, P3, Pz, and P4). Finally, during the test phase, the mean CNV amplitude was measured within 500–1,000 ms, and further analyses were performed at 500–700 ms and 700–1,000 ms, respectively. Repeated-measures ANOVA was performed considering action mode (intention-based and stimulus-based), anterior-posterior scalp location (anterior, medial, and posterior) and scalp laterality (left, middle, and right) as within-subject factors and age group (young and old) as a between-subject factor. Analysis of the congruent and incongruent task subgroups was conducted exclusively for the CNV within each age group, considering the task (congruent and incongruent) as a between-subject factor. Trials classified as incorrect were excluded from ERP analysis. The Greenhouse–Geisser correction was used to adjust for sphericity violations. *Post hoc* analysis for significant main effects was performed using the Bonferroni method when needed. Significant interactions were analyzed using a simple effects model.

Given the potential relationship between associative memory and bidirectional action-effect binding (Elsner et al., 2002; Melcher et al., 2008, 2013; Pfister et al., 2014), we also computed the correlations (Spearman’s rho) separately for the congruent and incongruent subgroups between the neuropsychological test scores and mean CNV amplitudes (i.e., the average of the CNV amplitude at the Cz, CPz and Pz electrodes) in each action mode and age group. The alpha level was fixed at *p* = 0.05 (two-tailed).

## Results

### Behavioral Results

Significant main effects of age [*F*_(1,34)_ = 5.98, *p* = 0.02, $\eta_{p}^{2}$ = 0.149] and action [*F*_(1,34)_ = 14.99, *p* < 0.001, $\eta_{p}^{2}$ = 0.306] were revealed in the analysis. The mean asynchrony of temporal bisection, as shown in **Figure 1B**, was larger (and earlier) for the old age group (-123 ms, *SE* = 17) than for the young age group (-66 ms, *SE* = 17), which indicates the inaccuracy of motor timing in older adults. Furthermore, the mean asynchrony of temporal bisection for stimulus-based action (-119 ms, *SE* = 14) was larger (and earlier) than that for intention-based action (-70 ms, *SE* = 12). These results confirmed the temporal attraction effect reported in previous studies (Waszak et al., 2005; Keller et al., 2006). The temporal attraction effect induced a time shift of a response toward its specific stimulus in stimulus-based actions, whereas the action that produced certain effects was shifted closer to its effect in intention-based actions. This effect revealed the mode difference between intention-based and stimulus-based actions, which demonstrates that both modes of action were performed well by the subjects according to the instructions provided to them in this study. The negative mean asynchrony value observed here has been commonly detected in synchronization tasks in previous studies (Aschersleben and Prinz, 1995). There was no significant action × age group interaction.

### ERP Results

#### Readiness Potential (-400–0 ms)

**FIGURE 2:**
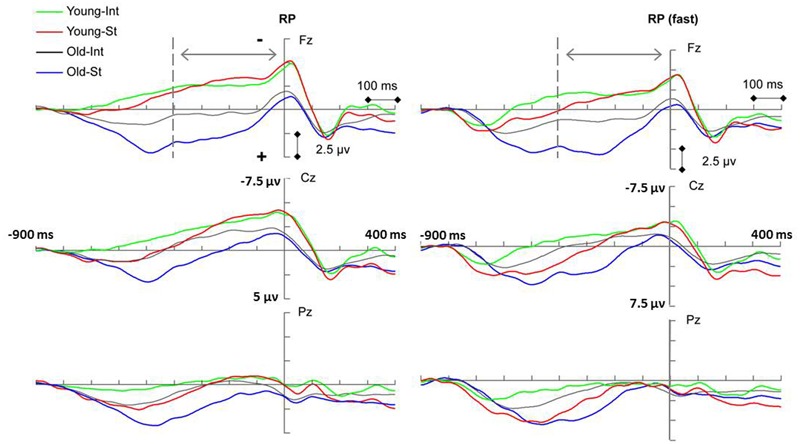
**Event-related potentials (ERP) waveforms showing significant main effect of age (*p* < 0.005) and action (*p* < 0.05) of readiness potential (RP) amplitude for both the undivided group (Left penal) and the fast-subgroup (Right penal).** INT, intention-based action; ST, stimulus-based action. Keypress response onset occurred at 0 ms.

The RP component reflected general movement preparation and the action-effect binding process in intention-based actions (Waszak et al., 2005; Keller et al., 2006). A late RP was used in this study (the 400 ms before movement onset, as suggested by Shibasaki and Hallett, 2006) because it is considered more sensitive to movement-related parameters than an earlier RP (Keller et al., 2006). **Figure 2** (left panel) presents the RP measurements for each condition. There was a significant main effect of age [*F*_(1,34)_ = 16.57, *p* < 0.001, $\eta_{p}^{2}$ = 0.328] and action [*F*_(1,34)_ = 6.54, *p* = 0.015, $\eta_{p}^{2}$ = 0.161], indicating that the RP amplitude of the two action conditions was significantly more negative for the young age group than for the old age group, which demonstrates the general age-related decline in the action preparation of both action modes. In addition, the RP amplitude for the intention-based condition was significantly more negative than that for the stimulus-based condition, which replicated the findings of previous studies with regard to the modal differences between the two actions. The interaction between action and age was also significant [*F*_(1,34)_ = 4.27, *p* = 0.046, $\eta_{p}^{2}$ = 0.112]. A simple effects analysis revealed that for the old age group, the RP amplitude for the intention-based condition was significantly more negative than that for the stimulus-based condition (*p* = 0.002), indicating that, although the RP amplitudes were decreased, modal differences between the two actions remained in the old age group. In contrast, these RP amplitudes did not significantly differ for the young age group (*p* = 0.73), which was inconsistent with previous studies. There was no significant interaction between either action or age and scalp location (*Fs* < 1.33, *p* > 0.27 for both).

In previous studies, modal differences in RP amplitude were observed among young subjects, specifically when different ISI durations were set between the two stimuli/effects. A 1,200 ms ISI was used in a study by Waszak et al. (2005), and ISIs of both 1,200 ms and 1,600 ms were used in a study by Keller et al. (2006) in which a smaller modal difference was observed for longer ISIs than for shorter ISIs. Therefore, considering the relatively long ISI (i.e., 1,600 ms) used in this study, further analyses were conducted on the young and old age groups whose RTs were shorter than average, classified herein as “fast subgroups.” The ERP trials were separated into two subgroups according to the mean RTs. For the “fast subgroup” analysis (see **Figure 2**, right panel), significant main effects were again found for both age group [*F*_(1,34)_ = 11.12, *p* = 0.002, $\eta_{p}^{2}$ = 0.246] and action [*F*_(1,34)_ = 30.05, *p* < 0.001, $\eta_{p}^{2}$ = 0.469]. There was also a significant interaction between action and age [*F*_(1,34)_ = 6.82, *p* = 0.013, $\eta_{p}^{2}$ = 0.167]. A simple effects analysis revealed a significant difference between the two action conditions for both the young (*p* = 0.05) and old (*p* < 0.001) “fast subgroups.” Thus, in this study, modal differences between two actions were observed for the young age group among the “fast subgroup” in which the trials corresponding to shorter than average RTs were pooled together. A possible explanation of the age effect is discussed later in this article.

#### N2 (300–400 ms)

**FIGURE 3:**
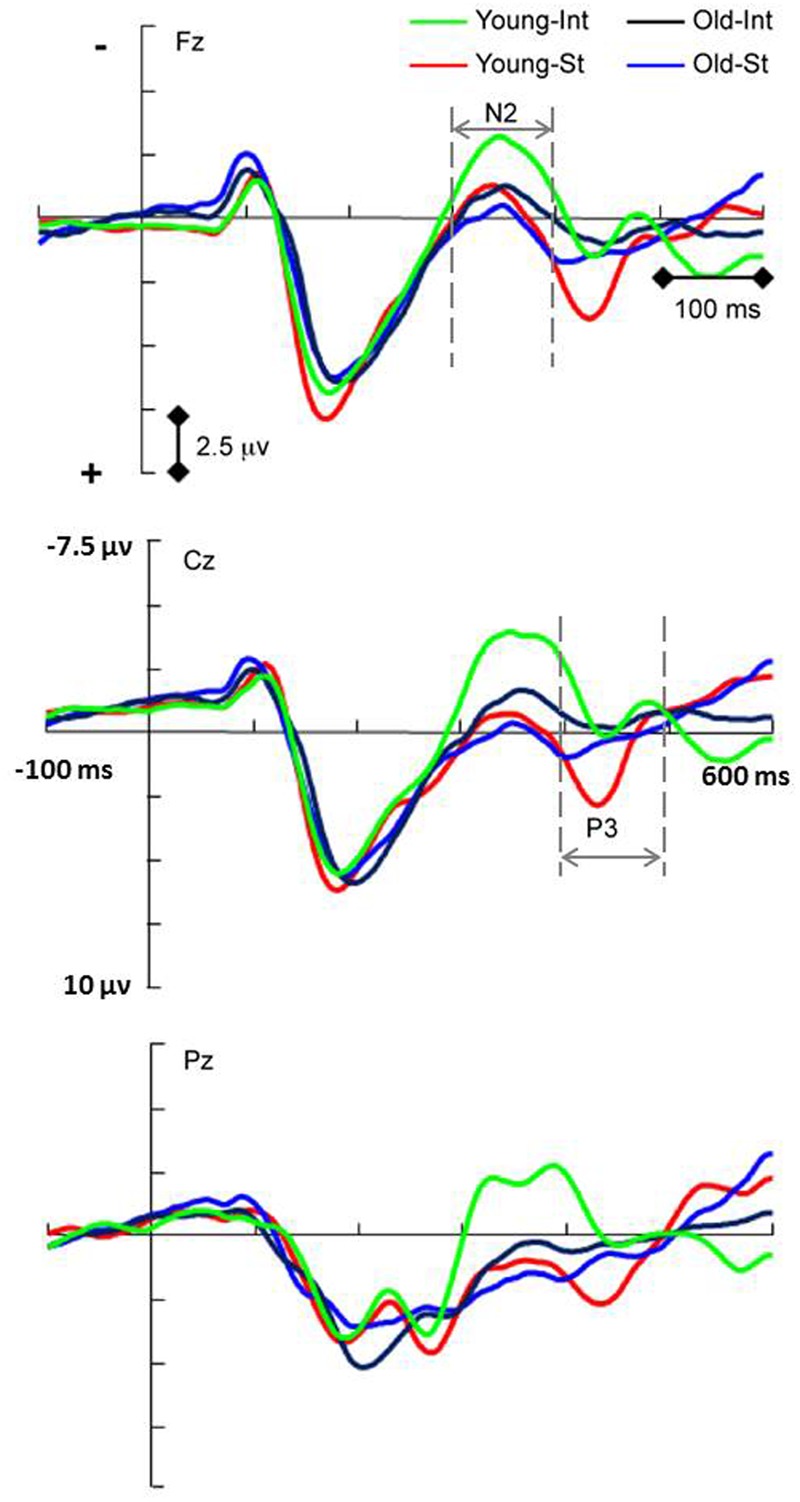
**Event-related potentials waveforms showing significant action × age interaction of N2 amplitude (*p* = 0.05) and P3 amplitude (*p* < 0.001).** Simple effect analysis showed the age effect of N2 in intention-based action (*p* = 0.007) and the age effect of P3 in stimulus-based action (*p* = 0.008). INT, intention-based action; ST, stimulus-based action. Visual stimulus onset occurred at 0 ms.

The N2 amplitude reflects the *feedback control* process during the acquisition phase in intention-based actions. For the analysis of the N2 component (**Figure 3**), there was no significant main effect of age. However, there was a significant main effect of action [*F*_(1,34)_ = 26.93, *p* < 0.001, $\eta_{p}^{2}$ = 0.442]: the N2 amplitude evoked by the intention-based condition was significantly more negative than that evoked by the stimulus-based condition, suggesting that compared with the stimulus-based action mode, the higher N2 amplitude was specific to the intention-based action mode. This finding is consistent with previous studies. The *feedback control* process functioned as a monitor to compare the actual action effect with the anticipated action effect that initiated the intention-based action (Hughes et al., 2013). An enhanced N2 component is observed when the actual action effect does not fit the expectation (Band et al., 2009), whereas an attenuated N2 component is observed when the actual action effect corresponds to the intended expectation (Hughes and Waszak, 2011). In this study, an attenuated N2 component should be detected because the actual action effect corresponded to the intended expectation. However, when comparing the two action modes, the N2 amplitude was more prominent for intention-based actions than stimulus-based actions.

There was also a significant interaction between action and age [*F*_(1,34)_ = 4.12, *p* = 0.05, $\eta_{p}^{2}$ = 0.108]. A simple effects analysis showed that the N2 amplitude of the intention-based condition was significantly more negative for the young age group than for the old age group (*p* = 0.007); however, the N2 amplitude for the stimulus-based condition did not significantly differ between the age groups (*p* = 0.62). This simple effect analysis also revealed an age-related decline in the *feedback control* process for intention-based actions, suggesting that this age effect was specific to certain processes and action modes.

Additionally, a significant interaction between action and scalp laterality was observed for the N2 component [*F*_(2,68)_ = 5.93, 𝜀 = 0.778, *p* = 0.008, $\eta_{p}^{2}$ = 0.149], showing that N2 amplitude difference between action modes was most pronounced in the middle line of electrodes than in the left or right line of electrodes. This was consistent with the study of Keller et al. (2006) that “the absence of lateralization of sensory areas” might be due to the centrally presented stimuli.

#### P3 (400–500 ms)

The P3 component was used in this study to reflect the *stimulus-response linkage* process during the acquisition phase in stimulus-based actions. There was no significant main effect of age for the P3 component. However, there was a significant main effect of action [*F*_(1,34)_= 77.55, *p* < 0.001, $\eta_{p}^{2}$ = 0.695], indicating that the P3 amplitude under the stimulus-based condition was significantly more positive than that under the intention-based condition (**Figure 3**). These findings replicate the modal differences between two actions reported previously (Waszak et al., 2005; Keller et al., 2006) when age groups were combined. There was also a significant interaction between action and age [*F*_(1,34)_ = 20.99, *p* < 0.001, $\eta_{p}^{2}$ = 0.382]. A simple effects analysis of this interaction showed that the P3 amplitude evoked by the stimulus-based condition was significantly more positive for the young age group than for the old age group (*p* = 0.008) but also that the P3 amplitude evoked by the intention-based condition did not differ between age groups (*p* = 0.141). This simple effects analysis revealed an age-related decline in the *stimulus-response linkage* process only for stimulus-based actions, suggesting that this age effect was specific to certain processes and action modes. There was also a significant interaction between action and scalp laterality for the P3 component [*F*_(2,68)_ = 8.74, *p* < 0.001, $\eta_{p}^{2}$ = 0.205], showing that P3 amplitude difference between action modes was most pronounced in the middle line of electrodes than in the left or right line of electrodes, which might be due to the centrally presented stimuli in this study. There were no other significant interactions between action or age and scalp location (all *F* < 0.93, all *p* > 0.35).

#### CNV (500–1,000 ms, 500–700 ms, or 700–1,000 ms)

**FIGURE 4:**
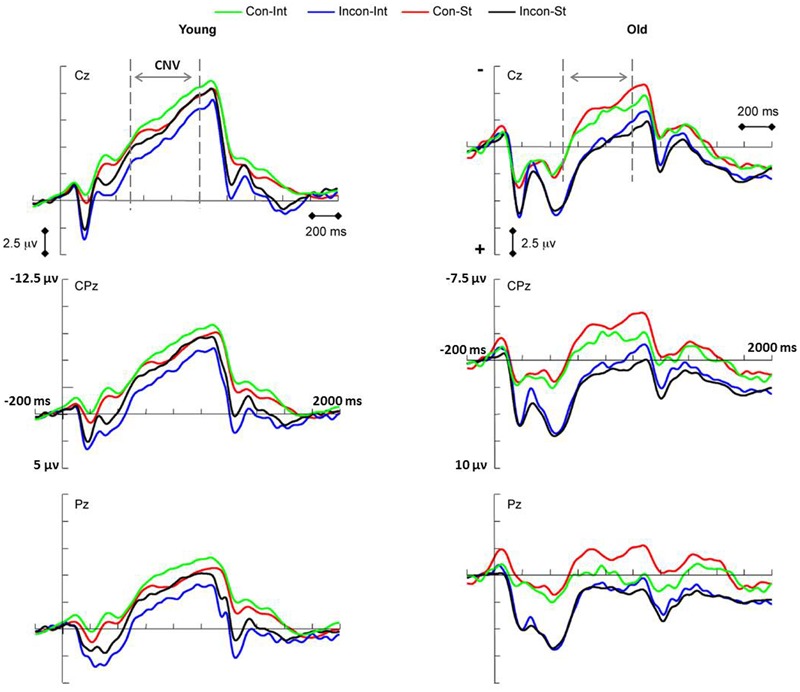
**Event-related potential waveforms showing the contingent negative variation (CNV) elicited by congruent (Con) and incongruent (Incon) tasks during the test phase of intention-based (INT) and stimulus-based (ST) actions in young (Left panel) and older (Right panel) adults.** A significant main effect of age (*p* < 0.001) was revealed when age groups combined. Left penal: young age group showing significant action × task interaction (*p* = 0.001). Simple effect analysis showed the interference effect of CNV amplitude in intention-based action (*p* = 0.045). Right penal: old age group showing marginally significant main effect of task (*p* = 0.051). The pre-cuing stimulus onset was set at 0 ms.

The CNV component was used to reflect the *effect-action retrieval* process during the test phase in intention-based actions. The CNV measurements for each condition are presented in **Figure 4**. There was a significant main effect of age [*F*_(1,34)_ = 23.54, *p* < 0.001, $\eta_{p}^{2}$ = 0.409] on the CNV amplitude (500–1,000 ms), which showed that the CNV amplitude of the two action conditions was significantly more negative for the young age group than for the old age group, demonstrating a general age-related decline in the movement preparation. There was no significant main effect of action or any interaction between age and action for the CNV amplitude.

In this study, the CNV component was recorded for a relatively long time period that included most of the negative deflection for the majority of the motor preparation components because all of the response information was provided by the appearance of a pre-cue. Thus, according to the instructions, the subjects prepared most of their movement response during the time period of the CNV amplitude measurement preceding action initiation. The interference effect of RT between congruent and incongruent tasks has been reported previously (Elsner and Hommel, 2001; Herwig et al., 2007) for intention-based action could be reflected within this period. The CNV amplitude has been linked to the sufficiency of motor preparation or the available resources to complete the task (Falkenstein et al., 2003; Leuthold et al., 2004). In Leuthold et al.’s (2004) study, the CNV amplitude increased with the amount of advanced information concerning movement preparation. Thus, we anticipated that there would be a significant difference in the amplitude of the CNV between congruent and incongruent tasks in intention-based actions in the young age group, specifically that the congruent task would elicit a more negative CNV amplitude than the incongruent task.

To investigate this interference effect of the CNV amplitude, two subgroups stratified by task congruency (congruent and incongruent task subgroups) were created for each age group. For the young age group, a significant interaction between action and task was found [*F*_(1,16)_ = 16.78, *p* = 0.001, $\eta_{p}^{2}$ = 0.512], although no significant main effect of either action or task was observed. A simple effects analysis revealed the interference effect of CNV amplitude as expected. The CNV amplitude evoked by the congruent task was significantly more negative than that evoked by the incongruent task under the intention-based condition (*p* = 0.045). However, under the stimulus-based condition, no significant difference in amplitude was observed between the tasks (*p* = 0.861). Thus, as we predicted, an interference effect of the CNV amplitude in intention-based actions was revealed in the young age group, demonstrating the intact function of the *effect-action retrieval* process among young adults. Specifically, the results showed that congruent *effect-action retrieval* might facilitate response preparation, whereas incongruent *effect-action retrieval* might interfere with response preparation.

However, this effect-action retrieval process might be impaired with age for the deficit of associative memory. For the old age group, there was no significant main effect of action or a task × action interaction, which demonstrates that the *effect-action retrieval* process of intention-based actions observed in the young age group was impaired in the old age group. However, there was a marginally significant main effect of task [*F*_(1,16)_ = 4.46, *p* = 0.051, $\eta_{p}^{2}$ = 0.218], which showed that under both action conditions, the CNV amplitude was more negative for the congruent task than for the incongruent task, suggesting that because of the age-related deficit, other cognitive processes might be involved. A possible explanation for this age effect is discussed later in this article.

Further analysis was conducted on the CNV amplitude within two smaller time windows. Consistent with the results of (500–1,000 ms) time window, significant main effect of age was found for (500–700 ms) time window [*F*_(1,34)_ = 24.40, *p* < 0.001, $\eta_{p}^{2}$ = 0.418] and (700–1,000 ms) time window [*F*_(1,34)_ = 19.07, *p* < 0.001, $\eta_{p}^{2}$ = 0.359], respectively, showing that the CNV amplitude was significantly more negative for the young age group than for the old age group. No significance was found for main effect of action or any interaction between age and action. Thus, a general age-related decline was observed continuously during the movement preparation.

The interference effect of CNV amplitude between congruent and incongruent tasks was also investigated within smaller time windows. For the young age group, the results were consistent with those of (500–1,000 ms) time window. Significant interaction between action and task was found within the 500–700 ms time window [*F*_(1,16)_ = 10.32, *p* = 0.005, $\eta_{p}^{2}$ = 0.392] and 700–1,000 ms time window [*F*_(1,16)_ = 18.66, *p* = 0.001, $\eta_{p}^{2}$ = 0.538], respectively, although there was no significant main effect of either action or task. Simple effects analysis revealed that, consistent with the results for the 500–1,000 ms time window, the CNV amplitude evoked by the congruent task was significantly more negative than that evoked by the incongruent task (500–700 ms: *p* = 0.028; 700–1,000 ms: *p* = 0.046) for the intention-based condition, whereas no significant difference was observed between tasks under the stimulus-based condition (500–700 ms: *p* = 0.504; 700–1,000 ms: *p* = 0.809). Thus, the interference effect of the CNV amplitude observed for the *effect-action retrieval* process in intention-based actions in the young age group remained consistent during the movement preparation period.

For the old age group, there was no significant main effect of action or action × task interaction within either the 500–700 ms and 700–1,000 ms time window, indicating that the interference effect of CNV amplitude observed in young age group was absent in old age group. However, a significant main effect of task condition [*F*_(1,16)_ = 6.74, *p* = 0.02, $\eta_{p}^{2}$ = 0.296] was found within 500–700 ms time window. This result, which is consistent with the marginal significance observed within the larger time window, indicates that the congruent task evoked a more negative CNV amplitude in both action modes during a relatively earlier period of movement preparation.

### Correlation Results

**FIGURE 5:**
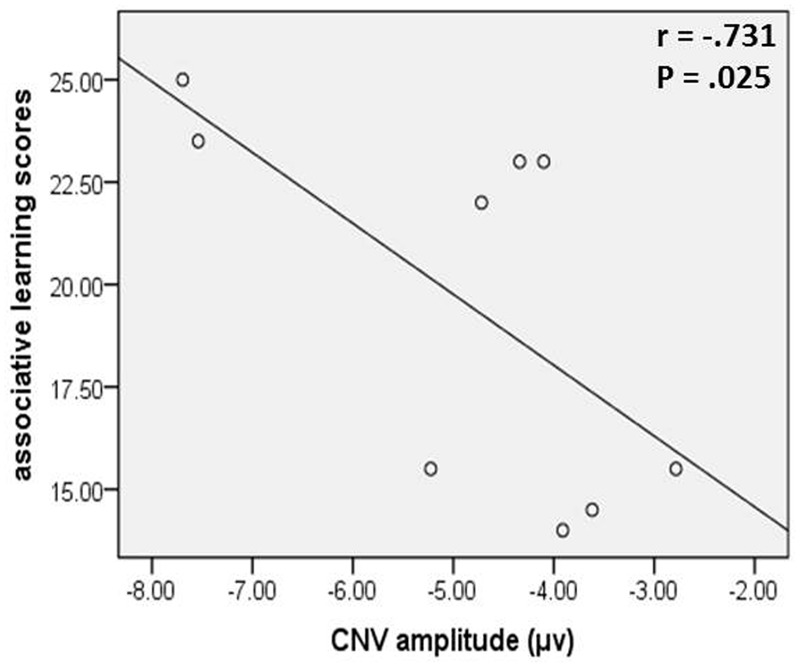
**The CNV amplitude of congruent task in intention-based action was negatively correlated with associative learning scores in young age group**.

In the young age group, associative learning scores, in which higher scores indicate a higher function level of associative memory, were negatively correlated with the CNV amplitudes (**Figure 5**), which were a negatively trending component, in the congruent task in intention-based action (*r_s_* = -0.731, *p* = 0.025). This result indicated a positive relationship between associative memory performance and motor preparation, as reflected by the CNV amplitude, which supports our assumption that associative memory might be, at least in part, responsible for the *effect-action retrieval* process in intention-based actions. However, no consistent evidence was observed in the incongruent task for the young age group or old age group. Therefore, further studies and more powerful evidence are needed to draw a cautious conclusion.

## Discussion

The aim of this study was to use ERP measurements to compare the age effects on four specific cognitive processes, namely *action-effect binding, stimulus-response linkage, feedback control*, and *effect-action retrieval*, between intention-based and stimulus-based actions. The age effects that were specific to these action modes and processes were thus detected. We observed a decrease in ERP amplitudes in the old age group with similar pattern as in the young age group during most specific processes. This result demonstrated a general age-related decline in action preparation. For the *effect-action retrieval* process in intention-based actions, we revealed an interference effect of the CNV amplitude in the young age group, which was absent in the old age group, indicating an age-related impairment on the *effect-action retrieval* of intention-based actions. One possible explanation for this finding might be the age-related decline in associative memory.

### Behavioral Temporal Attraction

In the behavioral results, the main effect of age showed larger mean asynchrony in the old age group than in the young age group, indicated age-related decline in motor timing accuracy. This age-related inaccuracy of motor timing might be influenced by both physical and neuromechanistic factors (Aschersleben, 2002). However, the absence of an interaction between action and age suggests that the action mode difference demonstrated by the temporal attraction effect still remained stable during the aging process, or at least in the early phase of aging, as represented by the subjects enrolled in this study.

### Action-Effect Binding

The RP component, a slow negative shift thought to reflect general movement preparation, is used to reflect the process of *action-effect binding* during the acquisition phase in intention-based actions (Waszak et al., 2005; Keller et al., 2006). The decreased RP amplitude observed for the old age group (compared with the young age group) demonstrated an general age-related decline during action preparation for both action modes. Specifically, when the action mode was considered, the previously reported modal differences in the RP (i.e., higher RP amplitude for intention-based actions than for stimulus-based actions) were observed only in the “fast subgroup” of young age group and with decreased amplitude in old age group, which was absent in young age group when fast and slow responses were combined together. These results suggested an age-related decline on movement preparation.

Given that reaction slowness in older adults has been consistently reported (e.g., Roggeveen et al., 2007; Berchicci et al., 2012; Wolkorte et al., 2014), the relatively long ISI of 1,600 ms utilized in this study seemed suitable for the old age group. However, the young age group may favor a faster response rhythm in the shorter ISI. When a slow response rhythm was demanded (as in this study), young participants might be able to intentionally allocate more resources than older participants within a prolonged period before movement execution due to some underlying mechanism. It has been emphasized that intentional and stimulus-related components might be involved in both action modes to varying extents (Keller et al., 2006; Herwig et al., 2007; Hughes et al., 2011) and that stimulus-based action might become less reflex-like and more intentional when the task becomes more complex or when delayed responses are needed (Herwig et al., 2007). From the grand average RP, we observed a trend in the young age group that the RP amplitude of stimulus-based action (much lower in the early period) increased rapidly in the later period to reach the RP amplitude of intention-based action, and this trend (rather than a reduction in intention-based RP amplitude) was responsible for the diminishment of the mode difference between the two actions in the young age group. Therefore, we suggest that the rapidly increased RP amplitude for stimulus-based action during the late RP period reflected the use of additional intentional efforts by young adults to hold corresponding responses for prolonged response periods. The absence of such a phenomenon in the old age group might suggest an insufficiency of available resources, which was also reflected by the decreased RP amplitudes for both action modes in the older age group than in the young age group. Interestingly, we previously observed in a training study that the RP amplitude of stimulus-based action (rather than of intention-based action) in healthy older adults was enhanced after receiving combined cognitive training (Niu et al., 2016), which was similar to the RP amplitudes in the young age group in this study, and might indicate that improved executive function facilitates the sufficiency of available resources. It should be noted, as Herwig et al. (2007) previously revealed, that the “less reflex-likeness” involving a greater intentional effort did not transform the stimulus-based action into an intention-based action. According to the ideomotor theory (Greenwald, 1970; Prinz, 1997; Elsner and Hommel, 2001; Prinz et al., 2009), intention-based actions are acquired/learned specifically through *action-effect binding* such that an action is performed with the anticipation of fulfilling a certain goal/effect. Therefore, it is necessary to investigate the age effect on separate action modes.

### Stimulus-Response Linkage

For the stimulus-based actions, a monodirectional *stimulus-response linkage* was formed during the acquisition phase and was reflected by the P3 components. An age effect was observed for stimulus-based but not intention based conditions, as the P3 amplitudes observed in the old age group were lower than those in the young age group. The P3 amplitude is traditionally considered to reflect attentional allocation during stimulus-related processing. In the examined action modes, P3 is thought to reflect the attentional process that helped form a linkage between a specific stimulus and its subsequent response (Petruo et al., 2016). Thus, P3 might serve as a marker reflecting completion of stimulus-related processing (Polich and Criado, 2006 for a review) and transformation of stimulus-related information into a corresponding response (Keller et al., 2006). As reviewed by Onofrj et al. (2001), cognitive P3 was most strongly influenced by age effects among all ERPs and was very sensitive to cognitive aging. In this study, the smaller P3 amplitude exhibited by the old age group under stimulus-based action suggested that limited stimulus-related resources were available to perform the action task compared with those available in the young age group. This observation is consistent with previous results showing that age-related declines of stimulus processing contributed to changes in movement with aging (Kolev et al., 2006; Sterr and Dean, 2008; Berchicci et al., 2012; Woods et al., 2015). For example, Berchicci et al. (2012) demonstrated that age-related slowing of stimulus processing correlated with the prolongation of both motor preparation and execution.

### Feedback Control

In this study, the *feedback control* process, as reflected by the N2 amplitude, was observed during the acquisition phase in intention-based actions rather than stimulus-based actions. The age effect, reflected by reduced N2 amplitude in the old age group relative to that in the young age group, was also observed for intention-based action only. This *feedback control* was considered to function as a monitor to compare the actual action effect with the anticipated action effect (Hughes et al., 2013), with a negative feedback mechanism for reinforcement learning (Holroyd and Coles, 2002). Thus, the age effect here suggested that older adults might be inadequately equipped with attentional resources and executive function for action effect monitoring or have impaired reinforcement learning based on negative feedback. Further studies are needed to explore this concept.

An argument might arise that the age effect we observed for the N2 and P3 amplitudes could reflect a general decline in attentional processing rather than a specific decline in pertinent action processing because these two components are related to attention allocation. If this was the case, the age effect should have been observed for both action modes. However, the main effect of age was not significant in this study for either the N2 or P3 component when two actions were combined. Instead, the age effect was revealed under a specific process that was peculiar to the action mode. The effect of age on the N2 amplitude reflecting the *feedback control* process was only observed in intention-based actions, whereas the age effect on the P3 amplitude reflecting the *stimulus-response linkage* process was only observed in stimulus-based actions. Therefore, as we assumed, we revealed the age-related declines peculiar to the specific action processes, which might be otherwise confounded under combined action modes or cognitive processes.

We can see that both RP and N2 are components reflecting cognitive processing of intention-based action, and these components occur successively in time. Therefore, the observed age effect on N2 amplitude might be influenced by the age effect on RP amplitude, as both components showed an enhanced amplitude in younger adults compared with that in older adults. However, a time interval (450–1,150 ms) between the execution of motor response and the onset of the following letter (i.e., correct trials) was set in this study, and this interval was longer than the 400-ms time delay suggested by Hughes and Waszak (2011). Based on this rather prolonged time interval, we believe that the N2 component data that we collected did not include an RP component. Moreover, as Onofrj et al. (2001) suggested in their review, age effects were usually reflected by decreased amplitudes of most ERP components. Thus, our findings of decreased amplitude for several ERPs during different processes during the acquisition phase suggest a general decline with age.

### Effect-Action Retrieval

During the test phase, the *effect-action retrieval* process was investigated using the CNV amplitude, where the age effects can be interpreted from two perspectives. First, the main effect of age resulted in decreased CNV amplitude in the old age group relative to that in the young age group, which demonstrated a general age-related decline in movement preparation. Second, distinct ERP patterns were revealed between the two age groups. The interference effect of CNV amplitude that was unique to intention-based action was absent in the old age group, and a main effect of task showing increased CNV amplitudes for congruent tasks relative to those for incongruent tasks on both action modes was observed within a smaller and earlier time window.

In the young age group, the observed interference effect of the CNV amplitude was consistent with previous behavioral reports (e.g., Elsner and Hommel, 2001; Herwig et al., 2007), which demonstrated the intact function of the *effect-action retrieval* process in intention-based actions. However, an absence of this interference effect was revealed in the old age group in this study and in another recent study (Niu et al., 2016) using the same paradigm in healthy older adults, suggesting an age-related impairment of the *effect-action retrieval* process. The *effect-action retrieval* in intention-based action has been considered a process of associative memory retrieval (Elsner et al., 2002; Melcher et al., 2008, 2013; Pfister et al., 2014), which was supported in this study by the correlation analysis in the young age group between associative memory performance and the CNV amplitude in congruent task in intention-based action. Associative memory is more sensitive to aging than other forms of memories (e.g., Naveh-Benjamin, 2000; see Yonelinas, 2002 for a review; Rhodes et al., 2008). Therefore, impaired associative memory might account for the absence of the interference effect of CNV amplitude in the old age group. According to the dual-process theory of memory, recollection (the retrieval of specific details) and familiarity (the general strength of information) are two main processes that control memory judgment. Recollection is particularly necessary to recognize rearranged pairs from learned pairs (recall-to-reject theory, Rotello and Heit, 2000; Gallo et al., 2004). For older adults, recollection is susceptible to aging, and familiarity may favor the retrieval of associative pairs. For example, older adults tend to recognize rearranged pairs as intact pairs with high scores of false alarm, particularly when the items that formed the pairs were repeatedly learned (Rhodes et al., 2008). In this study, action and effect pairs were repeatedly acquired and rearranged in the incongruent task. Older adults with recollection deficit may fail to recognize the rearrangement of action-effect pairs in the incongruent task and thus be unsusceptible to the interference caused by the incongruence. Therefore, the age-related deficit of recollection might account for the impaired *effect-action retrieval* in intention-based actions in older adults.

Interestingly, we also observed a main effect of task during the early part of motor preparation; older adults favored congruent tasks rather than incongruent tasks in both action modes, which could not be explained based on the evidence from associative memory. Some recent studies focusing on executive function of inhibition revealed an age effect on processing distractors (see Weeks and Hasher, 2014 for a review; Biss et al., 2013). It has been reported that processing distractors that are congruent with target goals could in fact facilitate the coding and retrieval of target stimuli for older adults. The possible explanations are that older adults are unable to inhibit the processing of irrelevant stimuli in the background and that congruent distractors aid the processing of target stimuli by increasing the depth of processing or implicit rehearsal. If these explanations hold true in the present study, executive function might be responsible for the main effect of task observed in older adults. However, it should be noted that executive function might represent a supplemental or compensative function when associative memory is impaired with age, since executive function has been suggested to play a fundamental and general role in action (see Colcombe and Kramer, 2003 for a review; Berchicci et al., 2012; Niu et al., 2016). The main effect of task occurred only during the early period of motor preparation and then disappeared when the motor response was about to be initiated. Further investigations are needed to understand the potential processes and mechanisms.

Some limitations should be noted in this study. First, the older adults enrolled in this study were less than 74 years of age and had high education levels and MMSE scores. These individuals may exhibit relatively intact cognitive function and reflect the age effect in an early stage. Further studies should include even older adults to evaluate how these age effects vary with the aging process. Second, behavioral performance and sufficient neuropsychological assessments should also be introduced for further validation. Third, during the test phase, a relatively small sample was examined in each subgroup, resulting in a large standard deviation, particularly in the old age group, and limiting the statistical power of the test hypothesis. Although the interference effect of CNV amplitude was observed with a small sample in the young age group, conclusions about the age effect must be made with caution. This pilot study provides a new perspective to investigate the age effect by comparing specific cognitive processes between different action modes. Further studies involving large sample sizes and multiple measurements are required to understand the age effects on action.

In summary, this study investigated the cognitive processes of two action modes from a developmental perspective and revealed the age effects between intention-based and stimulus-based actions via four cognitive processes: *action-effect binding, stimulus-response linkage, feedback control*, and *effect-action retrieval*. The results revealed not only generally declining functions of action preparation in older adults, as indicated by consistently decreasing ERP amplitudes, but also age effects specific to the action modes and processes, which might otherwise be mixed together under confounding experimental conditions. In particular, an interference effect indexed by the difference in the CNV amplitude between the congruent and incongruent tasks was observed in the young age group, which is consistent with previous behavioral reports. However, this effect was absent in the old age group, indicating an age-related deficit that is particular to the *effect-action retrieval* process of intention-based actions, which might be due to age-related deficits in associative memory. Further intervention studies targeting the improvement of intention-based action may add sufficient associative memory training to the executive function and physical exercise training.

## Author Contributions

Y-NN and JL conceptualized the design of the study. Y-NN researched the data and wrote the draft. XZ researched the data and contributed to the discussion. JL critically reviewed and edited the manuscript.

## Conflict of Interest Statement

The authors declare that the research was conducted in the absence of any commercial or financial relationships that could be construed as a potential conflict of interest. The reviewer JC and handling Editor declared their shared affiliation, and the handling Editor states that the process nevertheless met the standards of a fair and objective review.
